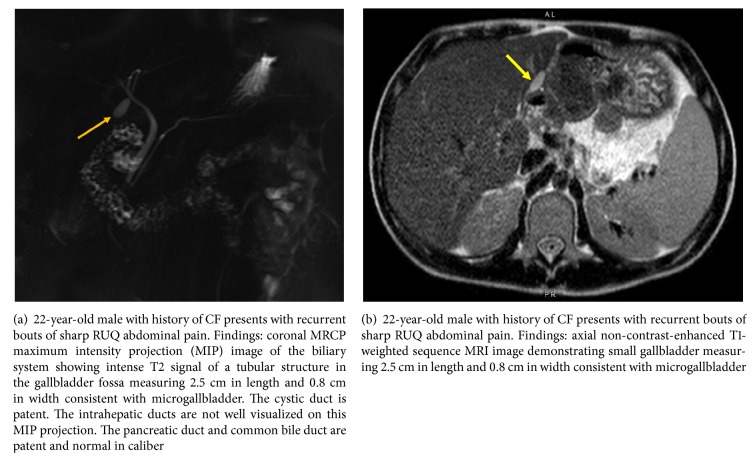# Microgallbladder: Self-Remitting Acute Cholecystitis-Like Condition Unique to Patients with Cystic Fibrosis

**DOI:** 10.1155/2019/6737428

**Published:** 2019-06-19

**Authors:** Mina S. Mousa, John C. Feldman, Paresh Mahajan

**Affiliations:** Department of Radiology, Morsani College of Medicine, University of South Florida, 12901 Bruce B. Downs Blvd., Tampa, FL 33612, USA

## Abstract

Microgallbladder is a nonsurgical medical condition characterized by chronic inflammation and atrophy of the gallbladder, which is considered a highly specific imaging finding unique to patients with cystic fibrosis (CF), and has been incidentally reported on abdominal imaging in up to 45% of cases with CF. The impairment of exocrine water efflux in CF leads to the production of hyperviscous biliary secretions, cholestasis, and transient cystic duct obstruction of the microgallbladder causing microcholecystitis—interestingly a self-remitting acute cholecystitis-like condition without surgical intervention. We present a case report of a 22-year-old male patient with history of CF with multiple hospital admissions for unexplained chronic abdominal pain found to be caused by microgallbladder, which was managed conservatively.

## 1. Introduction

Cystic fibrosis (CF) is an autosomal recessive genetic disease involving the cystic fibrosis transmembrane conductance regulator protein (CFTR), which plays a major role in regulating the secretory function and absorption of the respiratory, reproductive, and gastrointestinal systems, including the liver and pancreas [1–3]. Cystic fibrosis-related liver disease (CFLD) is a wide spectrum of disorders affecting the hepatobiliary system, which can lead to chronic gallbladder inflammation and atrophy, i.e., microgallbladder [4–6].

## 2. Case Presentation

**Figure 1:**
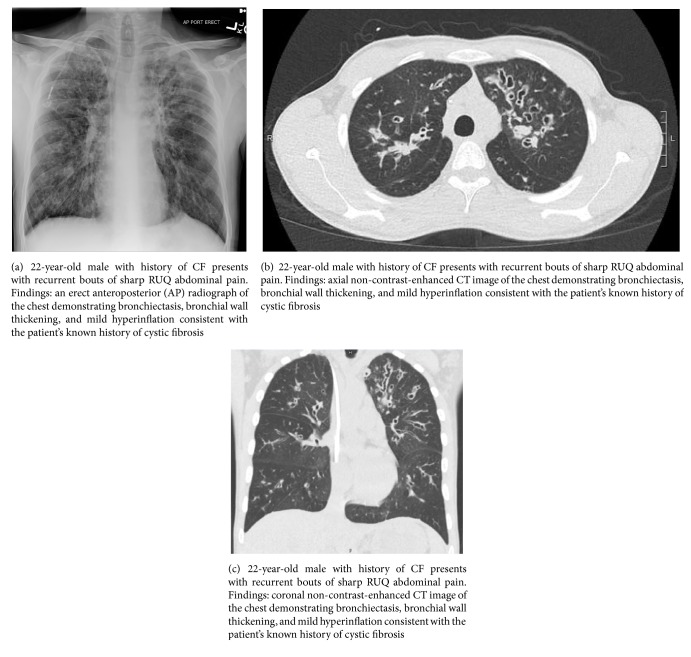


**Figure 2:**
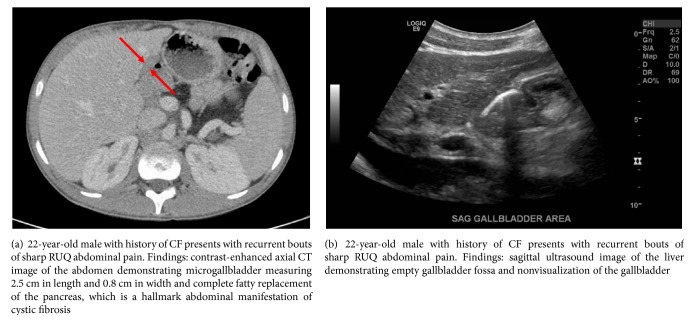


**Figure 3:**
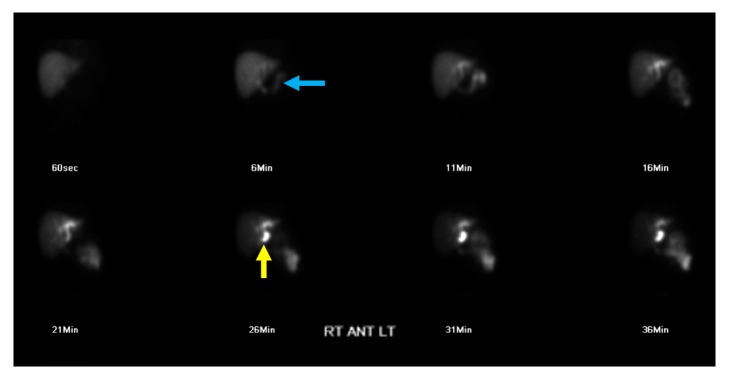
22-year-old male with history of CF presents with recurrent bouts of sharp RUQ abdominal pain. Findings: hepatobiliary scintigraphy HIDA scan demonstrating bowel activity (blue arrow) at 6 minutes and gallbladder activity (yellow arrow) at 26 minutes from the time of tracer intravenous injection of 3.3 mCi of Technetium 99m Choletec.

A 22-year-old male with history of cystic fibrosis without mention of meconium ileus presented to the emergency department for nausea, vomiting, subjective fever, and acute-on-chronic, self-remitting right upper quadrant (RUQ) abdominal pain for the past six years with no clear etiology, leading to multiple hospital admissions. Prior workup included esophagogastroduodenoscopy, colonoscopy, and laboratory and imaging studies (abdominal ultrasound and CT of the abdomen and pelvis), all of which were negative for underlying pathology, except for unexplained intermittent subjective fever, leukocytosis of 12,000–16,000 per uL, and RUQ sharp abdominal pain. Past medical history was otherwise unremarkable except for chronic exocrine pancreatic insufficiency due to CF, currently managed by oral pancrelipase medication. Past surgical history included laparoscopic appendectomy, with no prior history of cholecystectomy or history of cholelithiasis. During the current admission, the patient reported acute recurrence of nausea, vomiting, subjective fever, and sharp RUQ abdominal pain. Initial workup showed low-grade fever of 99-100°F, leukocytosis of 14,000 per uL, RUQ tenderness, and positive Murphy's sign on physical exam, similar to his prior hospital admissions. Other than low-grade fever, the remaining vital signs were within normal limits. Additional laboratory tests showed mildly elevated liver enzymes: alanine transaminase (ALT): 56–60 U/L, aspartate transaminase (AST): 35–76 U/L, alkaline phosphatase (ALP): 229–248 U/L, and gamma-glutamyl transpeptidase (GGT): 68 U/L. A chest radiograph and a non-contrast-enhanced chest CT demonstrated apical bronchiectasis with no signs of consolidation or pneumonia, unchanged when compared to the patient's prior studies (Figures 1(a)–1(c)). Prior abdominal CT and abdominal ultrasound (US) studies from the patient's previous admissions indicated nonvisualization of the gallbladder. On the abdominal CT study of the current admission, the gallbladder was not readily visualized; however, a small tubular structure in the gallbladder fossa measuring 2.5 cm in length and 0.8 cm in width raised the suspicion for gallbladder hypoplasia versus microgallbladder (Figures 2(a) and 2(b)). Subsequent hepatobiliary iminodiacetic acid (HIDA) scan (Figure 3) and magnetic resonance cholangiopancreatography (MRCP) demonstrated a small gallbladder with patent cystic duct corresponding anatomically to the tubular structure seen on the abdominal CT scan (Figures 4(a) and 4(b)). Due to the lack of imaging findings of gallstones, endoscopic retrograde cholangiopancreatography (ERCP) was not indicated at this time. After reviewing the literature, the diagnosis of microgallbladder was made based on the characteristic imaging findings of a small-size gallbladder and the patient's clinical history and presentation. The patient was treated conservatively with bowel rest and pain medication and was discharged on the third day of admission with outpatient follow-up.

## 3. Discussion

While the pathogenesis is not well understood, the etiology of microgallbladder is thought to be similar to exocrine pancreatic insufficiency: dysfunctional CFTR of the biliary exocrine tissues causing impairment of water efflux into the biliary system leading to hyperviscous secretions, i.e., biliary cholestasis and cholelithiasis [7–13]. Patients with this form of CF liver involvement may present clinically with self-remitting acute cholecystitis-like symptoms with or without jaundice, presumably due to the hyperviscous cholestasis causing transient cystic duct and/or common bile duct obstruction leading to inflammation of the microgallbladder, i.e., microcholecystitis [8, 9, 14].

The diagnostic criteria for microgallbladder are defined as less than 2–3 cm long and 0.5–1.5 cm wide on ultrasound evaluation [10, 15]. Since part of the diagnostic criteria for acute cholecystitis is distension of the gallbladder more than 8 cm in length and 4 cm in width, a normal gallbladder measurement should therefore range between 3–8 cm in length and 1.5–4 cm in width [16]. In a cohort study by Dietrich et al. (2002) of 72 patients with cystic fibrosis and 60 healthy subjects using the aforementioned ultrasound exclusion criteria, the incidence of microgallbladder was 25% (18/72) in patients with cystic fibrosis, versus 0% (0/60) in healthy individuals, which is in lieu of prior literature reports of microgallbladder incidence of 5–45% in patients with CF [10, 12, 15, 17, 18]. In addition to the imaging diagnostic criteria, the diagnosis of microgallbladder has to be taken in clinical context of known history of recurrent bouts of acute cholecystitis-like symptoms, due to the prevalence of microgallbladder mimickers in patients with CF and animal models with CFTR gene mutation, such as congenital gallbladder hypoplasia, gallbladder agenesis, and biliary atresia [1, 4, 19, 20].

In patients with CF, the presence of a microgallbladder is considered an ancillary sign for cystic fibrosis liver involvement [6, 8]. Cystic fibrosis liver involvement or cystic fibrosis-related liver disease (CFLD) is a wide spectrum of disorders involving the hepatobiliary system, which is considered the third most common cause of mortality in patients with CF, preceded by pulmonary disease and lung transplant complications, and is manifested in one-third of all patients with CF [21, 22]. The presumed pathophysiology of CFLD is similar to the progression of primary sclerosing cholangitis: chronic cholestasis and cholelithiasis causing recurrent bouts of ductal inflammation and fibrosis and eventually cirrhosis with or without portal hypertension [7, 8, 10, 21, 23]. In noncirrhotic CFLD, the predominant clinical manifestations are at least one of the following: (1) elevation of AST, ALT, and GGT more than twice the upper limit of normal, (2) hepatic steatosis (liver parenchyma hyperechogenicity and poor penetration on US, decreased attenuation on CT, or signal dropout on chemical shift MRI sequences), (3) hepatic fibrosis (histologic diagnosis), or (4) cholangiopathy (beading and strictures of the biliary system on US, MRI, CT, or ERCP) [7, 8, 17, 21–23]. In advanced cases, CFLD can progress to focal biliary cirrhosis and multilobular diffuse cirrhosis (small-size liver, nodular hepatic contour, and coarse heterogeneous parenchyma) with or without portal hypertension (splenomegaly, abdominal varices, and ascites) [17, 18, 20, 24].

## 4. Conclusion

In summary, in patients with CF with unexplained recurrent RUQ abdominal pain and no known past surgical history of cholecystectomy, the nonvisualization of the gallbladder on an otherwise negative abdominal imaging study should prompt the search for a microgallbladder [4, 9, 10, 18, 25, 26]. This can be confirmed with HIDA scan or MRI with MRCP in equivocal cases [10, 12, 26, 27]. MRI and MRCP in particular can help in better visualization of microgallbladder, compared to CT or US, due to the characteristic T2 hyperintense fluid signal of the bile secretions [26, 27]. An additional advantage of MRCP over conventional imaging modalities is the evaluation for cholangiopathy, such as biliary beading and strictures, and also ruling out cholelithiasis and choledocholithiasis, which can be seen in up to 25% of patients with CF compared to 10–15% in the general population [18, 26, 27]. Alternatively, a positive HIDA scan in a patient with known history of CF should be evaluated in conjunction with additional cross-sectional imaging modalities to avoid false positive diagnosis of acute cholecystitis due to the prevalence of microgallbladder in this patient population [12, 27, 28]. In older studies, prophylactic treatment with ursodeoxycholic acid was recommended for CFLD to reduce cholestasis; however, more recent studies have shown no response of calcium bilirubinate stones to ursodeoxycholic acid treatment, and currently microgallbladder-induced abdominal pain is considered a self-remitting condition requiring no surgical intervention [11, 13, 14, 17].

## Figures and Tables

**Figure 4:**